# Insulin Receptor Isoform A Modulates Metabolic Reprogramming of Breast Cancer Cells in Response to IGF2 and Insulin Stimulation

**DOI:** 10.3390/cells8091017

**Published:** 2019-09-01

**Authors:** Veronica Vella, Maria Luisa Nicolosi, Marika Giuliano, Andrea Morrione, Roberta Malaguarnera, Antonino Belfiore

**Affiliations:** 1 Endocrinology, Department of Clinical and Experimental Medicine, University of Catania, Garibaldi-Nesima Hospital, 95122 Catania, Italy; 2 Department of Pathology, Anatomy and Cell Biology, Thomas Jefferson University, Philadelphia, PA 19107, USA; 3 School of Human and Social Sciences, “Kore” University of Enna, 94100 Enna, Italy

**Keywords:** insulin receptor isoform A, IGF2, IGF1R, metabolic reprogramming, aerobic glycolysis, metabolic flexibility, breast cancer

## Abstract

Previously published work has demonstrated that overexpression of the insulin receptor isoform A (IR-A) might play a role in cancer progression and metastasis. The IR has a predominant metabolic role in physiology, but the potential role of IR-A in cancer metabolic reprogramming is unknown. We aimed to characterize the metabolic impact of IR-A and its ligand insulin like growth factor 2 (IGF2) in human breast cancer (BC) cells. To establish autocrine IGF2 action, we generated human BC cells MCF7 overexpressing the human IGF2, while we focused on the metabolic effect of IR-A by stably infecting *IGF1R*-ablated MCF7 (MCF7*^IGF1R^*^-ve^) cells with a human IR-A cDNA. We then evaluated the expression of key metabolism related molecules and measured real-time extracellular acidification rates and oxygen consumption rates using the Seahorse technology. MCF7/IGF2 cells showed increased proliferation and invasion associated with aerobic glycolysis and mitochondrial biogenesis and activity. In MCF7*^IGF1R^*^-ve^/IR-A cells insulin and IGF2 stimulated similar metabolic changes and were equipotent in eliciting proliferative responses, while IGF2 more potently induced invasion. The combined treatment with the glycolysis inhibitor 2-deoxyglucose (2DG) and the mitochondrial inhibitor metformin blocked cell invasion and colony formation with additive effects. Overall, these results indicate that IGF2 and IR-A overexpression may contribute to BC metabolic reprogramming.

## 1. Introduction

The insulin receptor (IR) is a key regulator of development, growth and metabolism. In adult life, the IR has a crucial role in the regulation of glucose metabolism, lipids and proteins, especially in insulin target tissues, such as liver, muscle and adipose tissue [1]. The IR is expressed as two isoforms (IR-A and IR-B), and it is now widely accepted that the IR-B is the isoform predominantly expressed in adult tissues mediating the metabolic actions of insulin [1]. In contrast, the IR-A is considered the “oncofetal” isoform because of its involvement in fetal growth and development, and frequent re-expression at high levels in most malignancies, where IR-A plays a role in tumor progression and resistance to therapy [1,2,3,4,5,6,7]. Notably, the IR-A is a dual receptor for both insulin and insulin like growth factor 2 (IGF2), and when overexpressed in cancer cells may mediate the effects of locally produced, autocrine and/or paracrine IGF2 as well as the effects of circulating insulin, particularly in insulin-resistant patients with chronic hyperinsulinemia [8,9]. Thus, IR-A overexpression by cancer cells is a factor contributing to the increased cancer risk and worsen cancer prognosis in obese and diabetic patients [10,11,12,13].

Previous work has provided convincing evidence that IR-A overexpression plays a role in various phases of cancer development and progression by promoting mitogenic, pro-invasive and pro-angiogenic programs [1]. Moreover, IR-A overexpression in cancer cells is a major factor contributing to resistance to insulin growth factor 1 (IGF1) receptor (IGF1R) specific inhibitors, which are only marginally effective in cancer therapy when used as single therapy [14,15]. These findings are not surprising as IR-A and IGF1R have similar affinities for IGF2 [16,17,18,19]. In addition, IR isoforms form hybrid receptors with IGF1R, IR/IGF1R hybrids, that are often not inhibited by anti-IGF1R antibodies [20,21,22].

Many lines of evidence have recently suggested that dysregulated receptor tyrosine-kinase (RTK) signaling in cancer might be implicated in metabolic reprogramming, which is a hallmark of cancer that can be targeted by several therapies [23]. However, whether the IGF2–IR-A axis elicits a role in cancer metabolic reprogramming is not established. 

Cancer cells preferentially metabolize glucose through aerobic glycolysis rather than using oxidative phosphorylation (OxPhos) (the so-called Warburg effect). Glycolysis, which is enhanced by various oncogenic events, represents a low efficiency process in synthesizing ATP as compared to OxPhos and therefore cancer cells need to maintain high glucose uptake [24,25]. Conversely, glycolysis has several advantages, including the ability to provide cells with substrates for the synthesis of nucleic acids and fatty acids required for increased proliferation. However, recent studies have demonstrated that high glycolytic activity may coexist with efficient OxPhos. Indeed, mitochondrial functions and biogenesis activated by mitogenic signals and energetic stress are also implicated in cancer progression [26] and OxPhos is required for cell migration, metastasis and resistance to TK inhibitors (TKI) [27]. Moreover, cancer cells have the ability to switch from glycolysis to OxPhos and vice versa (metabolic flexibility), which is a remarkable mechanism of adaptation to the different microenvironments favoring cancer dissemination.

In human breast cancer (BC), the IR and particularly the oncofetal IR-A isoform, is well expressed in approximately 40% of cases and markedly overexpressed in approximately 20% of cases [28,29,30]. Notably, approximately 20–40% of BCs occur in obese and/or type 2 diabetic patients exposed to high levels of circulating insulin [31]. Accordingly, constitutive IR autophosphorylation detectable in human BCs is likely explained by exposure to hyperinsulinemia and/or to local IGF2 secretion [32,33] and is associated with poor patient survival [34]. The IR homolog IGF1R is also expressed in 39% to 93% of human BC [35] and, although believed to contribute to breast oncogenesis, its prognostic role is unclear [36,37]. 

In this study, we hypothesized that autocrine IGF2 as well as IR-A overexpression might play a role in metabolic reprogramming of BC cells. Indeed, we found that MCF7 cells engineered to overexpress IGF2 showed increased proliferation and invasion with a concomitant increase in aerobic glycolysis but also in mitochondrial biogenesis and activity. Both extracellular acidification rates (ECAR), representative of glycolysis, and oxygen consumption rates (OCR), representative of OxPhos, increased under stressful conditions. Similar findings were observed in MCF7 cells lacking IGF1R and overexpressing solely the IR-A in response to either insulin or IGF2. Overall, these results indicate that IR-A overexpression/overactivation may contribute to BC metabolic reprogramming and increased metabolic flexibility.

## 2. Materials and Methods

### 2.1. Materials

Bovine serum albumin (BSA) and fibronectin were from Sigma-Aldrich (Saint Louis Missouri, USA); Metafectene PRO from Biontex Laboratories GmbH (Germany); Opti-Minimum Essential Medium (MEM, fetal calf serum (FCS), Puromycin, TRIzol Reagent, ThermoScript RT kit, SYBR Green MasterMix from Life Technologies, Inc. Laboratories (Paisley, UK); methyl thiazolyl tetrazolium (MTT), nitrocellulose membranes, horseradish peroxidase (HRP)-conjugated secondary antibodies were from Amersham Biosciences (Little Chalfont, UK); LY294002 and PD98059 were from Calbiochem (Darmstadt, Germany); OSI-906 was from Selleckem (Munich, Germany). Glucose Uptake Colorimetric Assay Kit (#K676-100) and Lactate Colorimetric Assay Kit II (#K627-100) were from BioVision, Inc. (Milpitas, CA, USA), MitoTracker™ Orange CMTMRos (#M7510) from ThermoFisher Scientific. Constructs encoding either an empty vector (pCMV6-Entry with C-terminal Myc-DDK Tag), or the human insulin like growth factor 2 (IGF2 Myc-DDK Tag) cDNAs were purchased from OriGene (Rockville, MD, USA). 

### 2.2. Cell Cultures 

The human cancer cell line MCF7 was purchased from the American Cell Type Culture Collection and cultured according to the manufacturer’s instructions. Cells were grown in complete MEM (Sigma, St. Louis, MO, USA) supplemented with 10% fetal bovine serum (FBS). MCF7/empty vector (EV) and MCF7/IGF2 cells were generated by transfecting MCF7 cells with the pCMV6-Entry vector or the human IGF2 cDNA containing vector (IGF2 Myc-DDK Tag), respectively. After transfection, cells were selected in 1 µg/mL puromycin containing medium and subcloned. MCF7 Clustered Regularly Interspaced Short Palindromic Repeats (CRISPR)-Cas9, knock-out (KO)-IGF1R, and KO-IR were purchased from Applied Biological Materials (Richmond, BC, Canada).

### 2.3. Generation of Lentiviral Vector pLEX^G418^–EV and pLEX^G418^–hIRA–FLAG 

Lentiviral vectors: pLEX–pCMV–IRES–PAC (PAC: puromycin resistance gene) lentiviral vector with cytomegalovirus constitutive promoter (pCMV) was used to obtain pLEX-pCMV-IRES-G418 (G418: geneticin resistance gene) as follows. We purchased the DNA sequence containing internal ribosome entry site (IRES) sequence conjugated with G418 obtaining IRES–G418 with NotI and HpaI restriction sites inserted at 5′ and 3′ positions respectively from Integrated DNA Technologies (Integrated DNA Technologies, Inc. Coralville, USA). pLEX–pCMV–IRES–PAC was subjected to enzymatic digestion by NotI and HpaI to replace IRES–PAC with IRES–G418 sequence obtaining pLEX–pCMV–IRES–G418 (pLEX^G418^–EV). To generate pLEX^G418^–*hIRA*–FLAG lentiviral vectors we amplified IR-A (*hIRA* v2, GENE ID: NM_001079817) and cDNA from RG215257-hIR-A plasmid (all from Origene) with the following primers containing FLAG sequence in the reverse primer: forward (Fw) *hIRA*: *5′-GGACTAGTGCCACCATGGCCACCGGGGGCCG-3′*; reverse (Rv) *hIRA*: *5′-CGACGCGTTTACTTATCGTCGTCATCCTTGTAATCGGAAGGATTGGACCGAGGC-3′*. hIRA–FLAG was cloned in pLEX^G418–^EV in SpeI and MluI restriction sites.

### 2.4. Lentiviral Particle Production and Lentiviral Transduction and Generation of MCF7-KO^IGF1R^-IR-A-FLAG

Recombinant lentiviruses were produced by transient transfection in T Large Antigen (TLA)-HEK-293t using the calcium phosphate method according to the protocol provided by Dharmacon. The lentiviruses supernatant was concentrated as previously described [38] and then titrated using Lenti-X p24 Rapid Titer Kit (TakaraBio) according to the manufacturer’s protocol. We used a multiplicity of infection (MOI) equal at five for MCF7-KO*^IGF1R^*. All cells were lentivirally transduced by spinoculation at 1200 g, 32 °C for 90 min in presence of 8 µg/mL of polybrene for two times each 24 h. At this time fresh medium was replaced, and cells cultivated for additional 48 h. Since MCF7-KO*^IGF1R^* cells were already resistant to puromycin we modified pLEX–pCMV–IRES–PAC lentiviral vector to obtain pLEX–pCMV–IRES–G418 as reported above. MCF7-KO*^IGF1R^* cells (50,000/cm^2^) were transduced using lentiviral particles containing pLEX^G418^–EV, pLEX^G418^–*hIRA*–FLAG, as indicated above. Cells were selected in 800 µg/mL of G418 for six days replacing G418 containing fresh medium every three days. At this time the cells were cultivated for additional six days in the absence of antibiotics and then exposed to puromycin plus G418 for additional three days. Viable cells were expanded in antibiotic-free medium, lysed and protein lysates subjected to immunoblot with anti-FLAG to detect the correct transgene expression.

### 2.5. Western Blot Analysis

For time-course experiments, sub-confluent cells were incubated in serum-deprived medium for 24 h and then solubilized in radioimmune precipitation (RIPA) buffer for the indicated time points. To evaluate IGF2-dependent activation, cells were serum-starved for 60 h and cell lysates were subjected to Western blot analysis as previously described. The following antibodies were used: anti-IRβ (C-19, sc-711) (Santa Cruz Biotechnology); anti-phospho(p)IGF1R (Tyr1135/1136)/pIR (Tyr1150/1151) (19H7), anti-p-Akt8 virus oncogene cellular homolog (Akt) (Ser473), anti-AKT, anti-p-extracellular signal-regulated kinase (ERK)1/2 (T202/Y204), anti-ERK1/2 (Cell Signaling Technology); anti-pIR (Y1334), specific for IR (Invitrogen), anti-lactate dehydrogenase A (LDHA) (216-228, SAB1100050), anti-lactate dehydrogenase B (LDHB) (AV48210), anti-pyruvate kinase M2 (PKM2) (isoform M1, SAB4200094), anti-βactin (Sigma Aldrich). 

### 2.6. Real-Time PCR and Mitochondrial DNA Copy Number

Total cellular RNA was extracted using TRIzol Reagent according to the manufacturer’s protocol. qRT-PCR was used to confirm the expression levels of mRNAs. Total RNA (2 μg) was reversely transcribed using the ThermoScript RT (Invitrogen) and oligo deoxy-thymidine (dT) primers. Synthesized cDNA was combined in a qRT-PCR reaction using primers for the gene of interest (Table 1). 

Real-time PCR was performed with an ABI 7500 Real-Time PCR System (Applied Biosystems) using probe, primer sets and SYBR Green chemistry. Human glyceraldehyde 3-phosphate dehydrogenase (GAPDH) and β-actin were used for normalization in SYBR Green chemistry. mRNA quantification was performed using the comparative cycle threshold (CT) method (ΔΔCt). For the real-time PCR estimation of the mitochondrial DNA (mtDNA) copy number total DNA samples were prepared using the cell DNA Isolation Kit (#53100, Norgen Biotek Corp). The mtDNA copy number was estimated by amplifying a portion of the tRNAleu of the mtDNA and comparing it to the amplification profile of a nuclear single copy gene, GAPDH.

### 2.7. IR Isoform mRNA Expression

IR isoform mRNA expression was measured by RT-PCR analysis (Bioline PCR Kit) using primers for the flanking exons 10 and 12 and resolved on 2.5% agarose gel. The 167-base pair (bp) and 131-bp DNA fragments, representing Ex11+ (IR-B isoform) and Ex11− (IR-A isoform), respectively, were quantified by densitometry analysis. The proportion of IR-A was calculated as densitometric value of band IR-A/densitometric values of bands IR-A + IR-B. 

### 2.8. Cell Proliferation

Cell proliferation was evaluated by cell counting after 24, 48, 72 and 96 h, using trypan blue exclusion of dead cells. Briefly, cells were seeded in 48-well plates in triplicates and grown in medium containing 2.5% of charcoal stripped-FCS. After 24 h cells were trypsinized and counted every 24 h as indicated in Results. 

### 2.9. Migration Assay

To measure migration, cells were seeded in six-wells plates to near confluency. After 24 h cell monolayers were scratched using a sterile p20 tip (time 0 h). Cells were allowed to migrate into the wound for 16–24 h. Pictures were taken of the wound at 0 and 24 h using the ×10 objective. The wound areas were analyzed using the following formula: Wound area (% of control) = (wound area after the indicated period/initial wound area) × 100 [39].

### 2.10. Invasion Assay

The ability of cells to invade the extracellular matrix was measured in Boyden’s chamber, as previously described [40]. Cells were seeded and, after 24 h, serum starved for further 24 h, removed from plates with 0.01% trypsin and placed on polycarbonate filters (8 μm pore size, Corning Costar), coated with 25 μg/mL fibronectin. After 6 h of incubation, cells on the upper surface of filters were removed with a cotton swab and filters were stained for 30 min with crystal violet (0.05% crystal violet in phosphate buffered saline (PBS) plus 20% ethanol). After three washes with water, crystal violet was solubilized in 10% acetic acid for 30 min at room temperature, and its concentration was evaluated by absorbance at 595 nm. 

### 2.11. Soft-Agar Colony Formation 

Anchorage-independent growth was assessed as previously described. Briefly, a mixture of 0.66% agar and medium containing 2.5% of charcoal stripped (CS)-FCS was plated on the bottom of each well plate (hard-agar). Then, cells suspended in 2.5% CS-FCS medium containing 0.33% agar (soft-agar) were plated on the top of the hard-agar layer. Top agar was then covered with culture medium. Cells were cultured for three weeks in the presence or absence of ligands, as indicated. Colonies were visualized with 0.5 mg/mL using methyl thiazolyl tetrazolium (MTT), photographed and analyzed with NIH ImageJ.

### 2.12. Glucose Uptake and Lactate Production

MCF7/EV and MCF7/IGF2 cells were seeded into six-well plates at 3 × 10^5^ cells per well. Cell culture medium was collected and frozen in −80 °C, and cell number was measured with hemocytometer. Glucose uptake and lactate production were determined using a Glucose Assay Kit (BioVision, Inc., San Francisco, CA, USA; K686) and Lactate Assay Kit (BioVision, K627), respectively, according to the manufacturer’s instructions. 

### 2.13. Mitochondrial Staining and Flow Cytometry 

Mitochondrial activity was evaluated incubating cells with 25 nM of MitoTracker Orange (#M7510, Molecular Probes, Invitrogen) probe. Briefly, cells were seeded in six-well plate at a density of 3 × 10^5^ cells per well; 60 h later cells were incubated in the presence of the relevant probe for 30 min at 37 °C in the dark. Cells were then washed in PBS, trypsinized, and analyzed by fluorescence activated cell sorting (FACS) using FACSCalibur (BD Biosciences, Europe) and Cellquest Pro software (Beckton Dickinson). The fluorescence intensity of cells of 10,000 events was recorded. The geometric mean of three technical replicates of fluorescent cells was calculated. 

### 2.14. Glycolytic Function and Mitochondrial Respiration Assay

Cells were seeded in extracellular flux (XF)p cell culture microplates (Seahorse Biosciences, Santa Clara, CA, USA) at a density of 10^4^ cells per well. Then cells were treated with ligands as indicated and subjected to glycolytic function and mitochondrial respiration assay using an XFp Extracellular Flux Analyzer (Seahorse Biosciences) according to the manufacturer’s instructions. Indicated chemicals in ports of XFp Sensor Cartridge (Seahorse Biosciences, Santa Clara, CA, USA) were injected into each well at specified time points. ECAR and OCR values were recorded and normalized to cell number.

### 2.15. Densitometric and Statistical Analysis

Densitometry results were obtained by using NIH ImageJ. Differences between means were evaluated by one-way ANOVA followed by post-hoc analysis of significance (Bonferroni test) for the comparison between more than two groups, whereas the Student’s *t* test for unpaired samples was used for comparisons between two groups. The level of significance was set at *p* < 0.05. Statistical analysis was performed with GraphPad Prism6 (GraphPad Software, San Diego, CA, USA). Data were expressed as means ± standard error of the mean (SEM).

## 3. Results

### 3.1. Establishment of Human BC Cells with Constitutive Autocrine IGF2 Secretion

**Figure 1:**
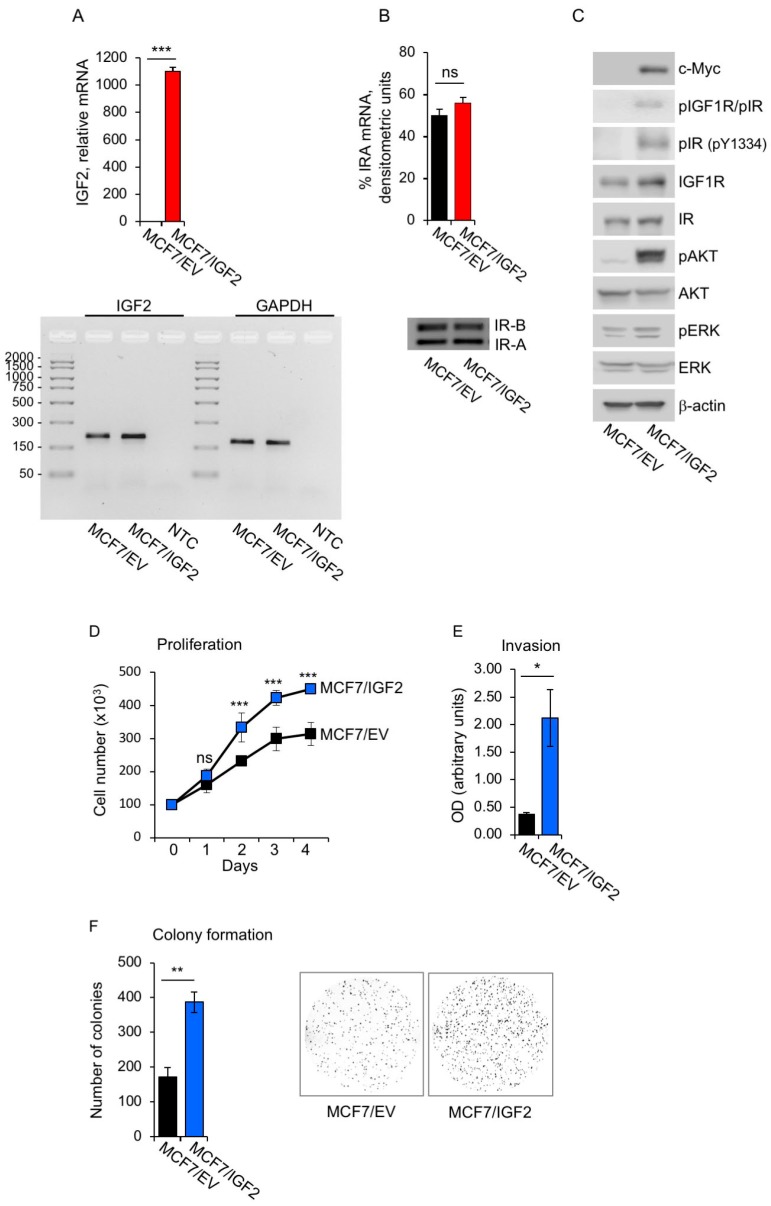
MCF7/insulin like growth factor 2 (IGF2) cells showed constitutively activated insulin receptor isoform A (IR-A), insulin growth factor receptor 1 (IGF1R), downstream signaling and increased protumorigenic behavior. (**A**) IGF2 mRNA levels were measured in MCF7/IGF2 and control MCF7/empty vector (EV) cells by qRT-PCR. Normalization was done using human β-actin as the housekeeping control gene. Data are presented as means ± standard error of the mean (SEM) (error bars) from three independent experiments and show very high levels of IGF2 in MCF7/IGF2 as compared to control cells (upper panel). A representative of three independent experiments is shown. (*** *p* < 0.001); analysis of IGF2 qRT-PCR products from MCF7/EV and MCF7/IGF2 cells is shown in the lower panel. Glyceraldehyde 3-phosphate dehydrogenase (GAPDH) was used as housekeeping control gene and no template control (NTC) as negative control. (**B**) IR isoform (IR-A and IR-B) transcripts were obtained from both MCF7/EV and MCF7/IGF2 cells. Products of PCR amplification were resolved on a 2.5% agarose gel. Images of PCR products from IR-B (Ex+11) and IR-A (Ex-11) are 167 and 131 base pair (bp), respectively. Graphical representation of PCR analysis indicated the percentage of IR-A mRNA calculated as follows: densitometric value of IR-A band/densitometric value of IR-A + IR-B bands. Scanning densitometry was performed using ImageJ software. All results are expressed as means ± SEM of three independent experiments. (**C**) Anti-phospho-(p)IGF1R (Tyr1135/1136)/pIR (Tyr1150/1151) detecting both pIR and pIGF1R and anti-pIR (Y1334), specific for pIR, were used to assess autophosphorylation of the two receptors. Anti-p Akt8 virus oncogene cellular homolog (Akt) (Ser473) and anti-p extracellular signal-regulated kinase (ERK)1/2 (T202/Y204), were used to measure the activation of both AKT and ERK1/2. β-actin was used as control for protein loading. Cells grown in 10% charcoal stripped-fetal bovine serum (FBS) were lysed and analyzed by SDS-PAGE and immunoblotted with the indicated primary antibodies. Myc blotting was used to determine myc-tagged IGF2 levels in transfected cells. Blot is representative of three independent experiments. (**D**) Cell proliferation. Cell number was measured by trypan blue exclusion assay at the indicated time points. Values are means ± SEM of three independent experiments done in duplicate and are expressed as percent of EV cells (considered as basal). (**E**) Cell invasion. Cells were seeded on polycarbonate filters coated with 25 µg/mL fibronectin and allowed to migrate for 6 h to the lower chamber. Values show means and range of two independent experiments done in duplicate. (**F**) Colony formation. Cells were seeded in soft-agar, as described in Methods, and grown in 5% charcoal stripped-serum for three weeks. Colonies were stained with methyl thiazolyl tetrazolium (MTT) and then photographed. The histogram on the right represents the number (mean and range) of colonies from two independent experiments, each run in quadruplicate wells. (ns, not significant; *** *p* < 0.001).

In order to generate human BC cells with constitutive autocrine IGF2 secretion, we stably transfected MCF7 cells with a myc-tagged IGF2 vector (MCF7/IGF2). MCF7 are estrogen receptor (ER) positive cells with ductal characteristics, expressing high levels of IGF1R and IR, and biologically responsive to stimulation with insulin, IGF1 and IGF2 [41]. MCF7/IGF2 cells expressed significantly higher IGF2 mRNA levels as compared to MCF7/EV cells (Figure 1A, upper panel), although the latter cells still expressed IGF2 transcript (Figure 1A, lower panel) [41]. We analyzed the relative abundance of the two IR isoforms and demonstrated that the IR-A accounted for 50–60% of total IR with no significant differences between MCF7/IGF2 and MCF7/EV cells (Figure 1B). Notably, MCF7/IGF2 cells showed constitutive phosphorylation of both IR and IGF1R [42] as revealed by phosphoantibodies recognizing both receptors and by a phosphoantibody specifically recognizing pIR at Y1334 [43]. Downstream AKT and ERK1/2 kinases were also clearly phosphorylated (Figure 1C). In contrast, MCF7/EV showed undetectable phosphorylation of IR and IGF1R, a very low degree of AKT phosphorylation and low ERK1/2 phosphorylation (Figure 1C). Overall, these data indicated that constitutive autocrine IGF2 activated the two major signaling pathways through activation of both IGF1R and IR-A. 

We then biologically characterized MCF7/IGF2 cells and demonstrated a significantly higher proliferation rate than MCF7/EV control cells (Figure 1D) and enhanced ability to migrate through fibronectin-coated filters (>500%) (Figure 1E). In addition, MCF7/IGF2 cells showed stronger growth in anchorage-independency (>300%) compared to MCF7/EV cells as demonstrated by measuring the ability to form colonies in semi-solid agar (Figure 1F). Taken together, these results indicated that autocrine secretion of IGF2 in human BC cells induced constitutive activation of signaling cascades downstream of the IR-A and IGF1R and enhanced growth and protumorigenic activities. 

#### MCF7/IGF2 Cells Showed Increased Glycolytic and Mitochondrial Activities 

**Figure 2:**
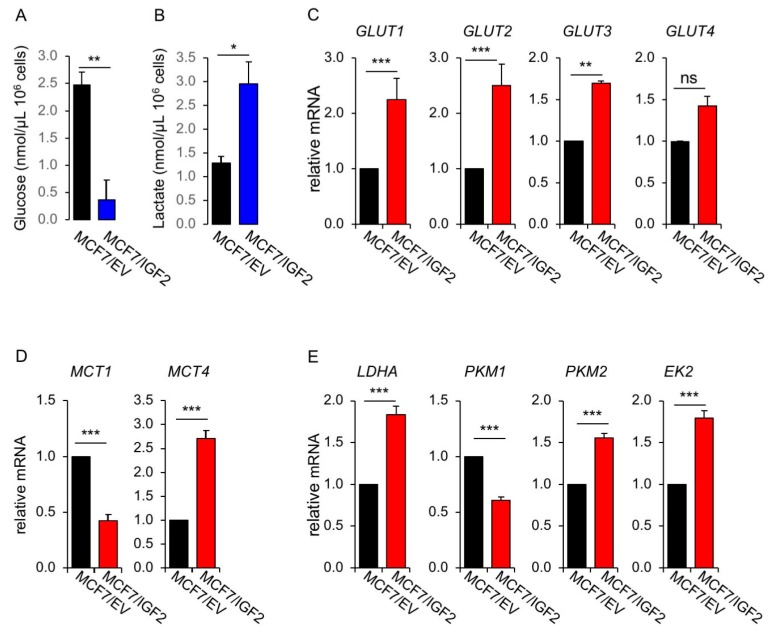

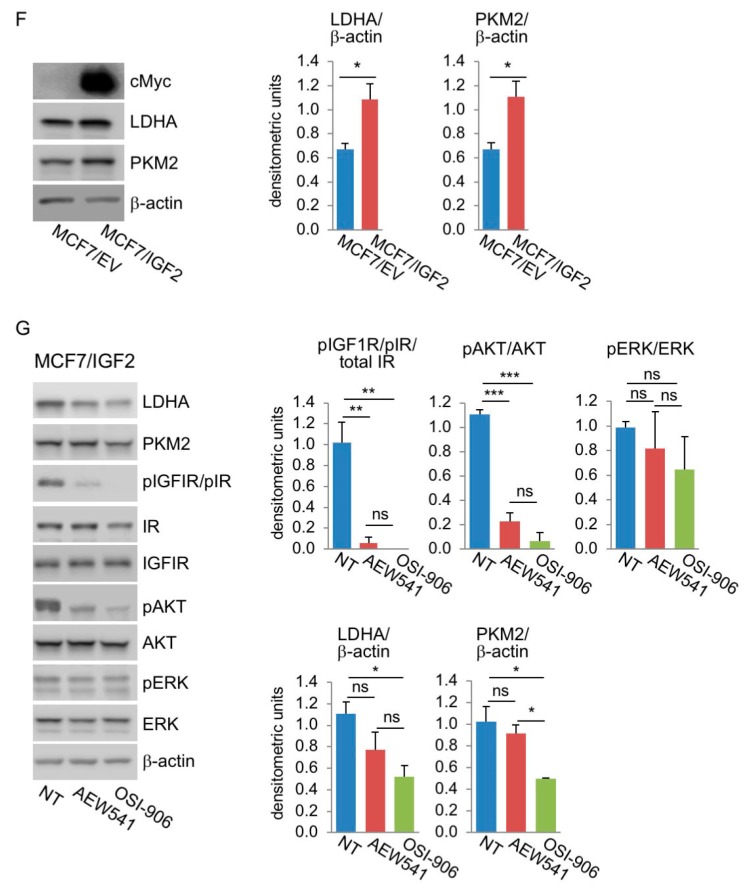
Glucose consumption, lactate production and expression of glucose and lactate transporters and glycolytic enzymes. Glucose (**A**) and lactate (**B**) concentrations were measured in media conditioned from MCF7/IGF2 and MCF7/EV control cells after 48 h using colorimetric assays (see Methods). Values are mean ± SE of three separate experiments (** *p* < 0.01; * *p* < 0.05). Cells were processed to evaluate mRNA expression for (**C**) glucose transporters (GLUT)1–4, (**D**) monocarboxylate transporter (MCT)1 and MCT4, (**E**) glycolytic enzymes lactate dehydrogenase A (LDHA), pyruvate kinase M1 (PKM1), pyruvate kinase M2 (PKM2), hexokinase-2 (EK2), by qRT-PCR analysis. MCF7/EV cells were used as control and GAPDH used as the housekeeping control gene. Values are means ± SEM of three separate experiments. (**F**) MCF7/IGF2 cells and the corresponding control cells (MCF7/EV) were grown in medium containing 10% charcoal stripped- fetal bovine serum (FBS) for 48 h. Cells were then lysed, analyzed by SDS-PAGE and immunoblotted with the indicated primary antibodies to evaluate the expression of glycolytic enzymes LDHA and PKM2. A representative blot of three independent experiments is shown. Graphs represent the mean ± SEM of densitometric analysis of three independent experiments, where LDHA and PKM2 were normalized to β-actin. (**G**) LDHA, PKM2, pIGF1R/pIR, IR, IGF1R, pAKT, AKT, pERK and ERK were then evaluated in MCF7/IGF2 cells before and after exposure to two different IGF1R/IR tyrosine-kinase inhibitors (TKI) (a pyrrolo(2,3-d) pyrimidine derivative, NVP-AEW541, and a selective and orally bioavailable dual IGF1R/IR inhibitor, OSI-906) at the concentration of 0.5 µM every 24 h, for 48 h. β-actin was used to control for protein loading. Blots are representative of three independent experiments. Graphs represent the mean ± SEM of densitometric analysis of three independent experiments.

1. Glycolytic activity. To assess whether constitutive IGF2 expression affects the metabolic phenotype of BC cells, we first evaluated glucose consumption, lactate production and the expression of transporters for glucose and lactate as well as key glycolytic enzymes in both MCF7/IGF2 and control MCF7/EV cells. MCF7/IGF2 cells, incubated for 48 h in serum free medium, showed reduced extracellular glucose levels (0.37 ± 0.37 nmol/µL vs. 2.47 ± 0.24 nmol/µL of glucose concentration per 10^6^ cells in MCF7/IGF2 and in MCF7/EV cells, respectively; *p* = 0.009) suggesting increased glucose uptake/consumption (Figure 2A). At the same time, MCF7/IGF2 cells showed increased lactate secretion in the conditioned medium (2.95 ± 0.47 nmol/µL vs. 1.29 ± 0.15 nmol/µL of lactate concentration per 10^6^ cells in MCF7/IGF2 and MCF7/EV cells, respectively; *p* = 0.028) (Figure 2B), suggesting increased lactate production.

Glucose transport into cells is regulated by membrane translocation of glucose transporters (GLUTs), therefore we evaluated the expression of GLUT1–4 (Figure 2C). MCF7/IGF2 cells showed higher mRNA expression levels for GLUT1, GLUT2, GLUT3 and GLUT4 as compared to control cells. Cancer cells largely relying on glycolysis produce huge amount of metabolic acids as end products and require the activity of monocarboxylate transporters (MCTs). MCTs are membrane carriers involved in transporting lactate, pyruvate and ketone bodies, and maintaining energy metabolism, homeostasis and pH control in tissues [23]. MCTs are often up-regulated in highly glycolytic tumors in order to counteract apoptosis driven by cellular acidosis. Thus, we assessed the expression of transporters for lactate such as MCT1, which supports lactate entry, and MCT4, which favors lactate cell efflux. MCF7/IGF2 cells showed lower (−27% ± 0.03%) expression of MCT1, as compared to control cells, while expressing higher expression levels (+271% ± 17%) of MCT4 (Figure 2D), suggesting a predominant requirement of MCT4 aimed at reducing intracellular lactate by promoting its efflux. We also measured the transcripts of key players of energy metabolism in glycolytic cells, such as LDHA, pyruvate kinase M1 (PKM1), PKM2, hexokinase-2 (Figure 2E). We discovered that MCF7/IGF2 expressed significantly higher levels of LDHA, PKM2 and hexokinase-2 expression as compared to control cells. LDHB isoform was present at very low level (not shown). Increased protein expression for LDHA and PKM2 were confirmed by Western blot analysis (Figure 2F). In order to confirm that this coordinated increase in key molecules involved in glycolysis observed in MCF7/IGF2 cells was dependent on IR/IGF1R TK activity, we exposed cells to two different IR/IGF1R TKI, a pyrrolo(2,3-d) pyrimidine derivative (NVP-AEW541) and a selective and orally bioavailable dual IGF1R/IR inhibitor (OSI-906, linsitinib), at the concentration of 0.5 µM every 24 h, for 48 h. As predicted, (Figure 2G) both TKI blocked IR/IGF1R autophosphorylation and downstream AKT activation, and reduced the levels of glycolytic enzymes LDHA and PKM2.

**Figure 3:**
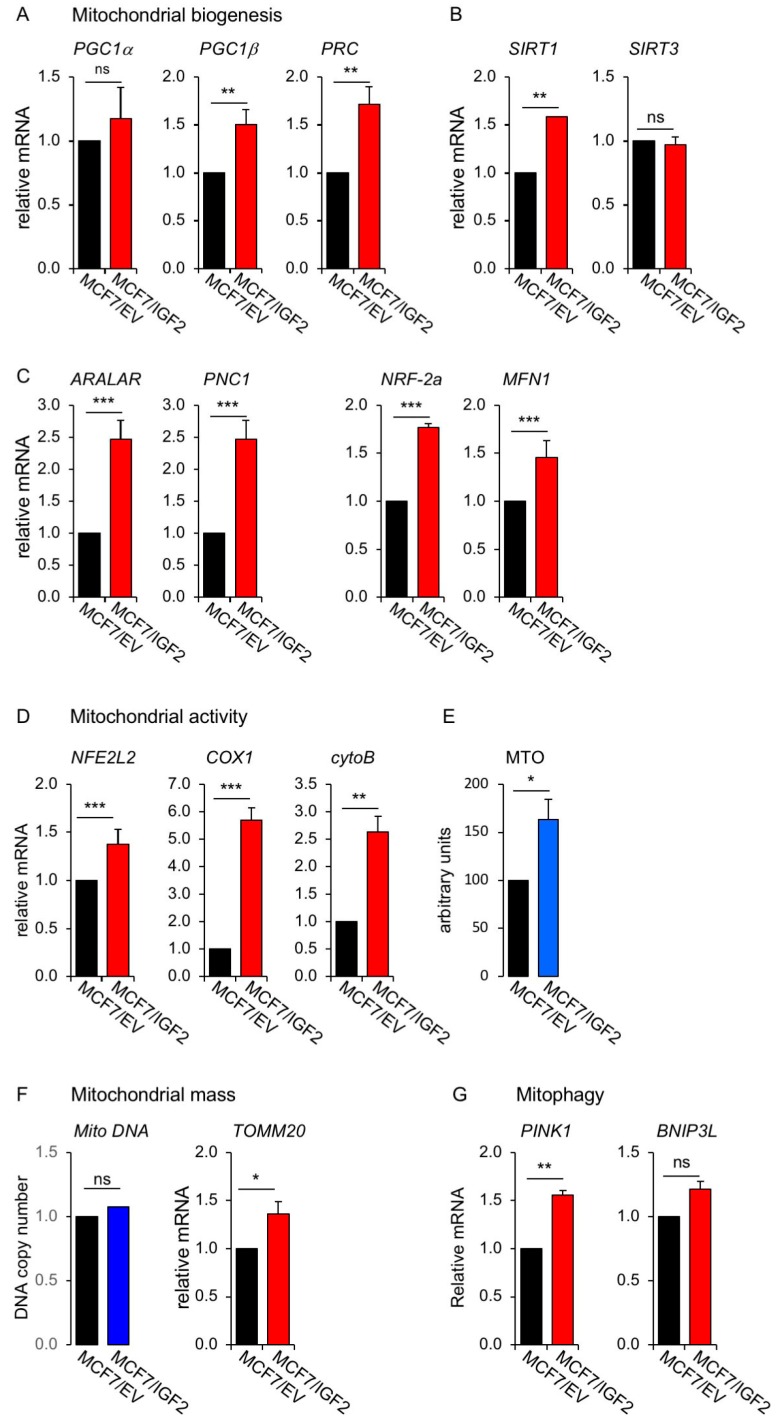
Markers of mitochondrial biogenesis and activity, mitochondrial mass and mitophagy were regulated in MCF7/IGF2 cells. (**A**) mRNA expression levels of mitochondrial genes, peroxisome proliferator-activated receptor gamma coactivator-1 (PGC1)α, PGC1β and PGC1α-related coactivator (PRC); (**B**) Sirtuin (SIRT)1 and SIRT3 mRNA expression; (**C**) aspartate–glutamate mitochondrial carrier 1 (ARALAR), pyrimidine nucleotide carrier 1 (PNC1), nuclear respiratory factor (NRF)-2a, and mitofusin (MFN)1 mRNA expression. (**D**) mRNA expression levels of mitochondrial activity genes nuclear factor erythroid-derived 2-like-2 (NFE2L2), cytochrome C oxidase 1 (COX1) and cytochrome B (cytoB). (**E**) Mitochondrial activity was evaluated by fluorescence activated cell sorting (FACS) analysis using MitoTracker Orange (MTO) probe. (**F**) Mitochondrial mass was assessed by measuring mtDNA copy number (left panel), and mRNA levels of mitochondrial outer membrane (TOMM20) (right panel). (**G**) Mitophagy mediators: mRNA expression levels of phosphatase and tensin homolog (PTEN)-induced kinase 1 (PINK1) and B-cell lymphoma 2 (BCL2)/adenovirus E1B 19 kDa protein-interacting protein 3-like (BNIP3L). MCF7/IGF2 and MCF7/EV cells were cultured in 2.5% charcoal stripped-FBS medium, and mRNA levels were determined by qRT-PCR. MCF7/EV cells were used as calibrator and GAPDH used as the housekeeping control gene in all qRT-PCR experiments. Values are means ± SEM of three independent experiments. (ns, not significant; * *p* < 0.05; ** *p* < 0.01; *** *p* < 0.001).

2. Mitochondrial function. In order to evaluate mitochondrial function in MCF7/IGF2 cells, we first investigated the expression of the peroxisome proliferator-activated receptor gamma coactivator-1 (PGC1) family of transcriptional co-activators, involved in mitochondrial biogenesis [24,25]. We found a significantly higher expression of PGC1α, PGC1β and PGC1α-related coactivator (PRC) in MCF7/IGF2 cells as compared to control cells (Figure 3A). We observed also higher levels of Sirtuin 1 (SIRT1) (Figure 3B), a deacetylase that increases mitochondrial biogenesis and OxPhos by activating PGC1α in nutrient deprivation conditions [26] and stimulating mitophagy [27]. On the contrary, SIRT3 was not affected. Elevated PGC1α levels can confer metabolic flexibility to cancer cells allowing the switch between mitochondrial and glycolytic metabolism for ATP production [28]. Moreover, mitochondrial biogenesis requires the expression of genes encoded by both mitochondrial and nuclear genomes under the control of PGC1 isoforms and activated by mitogenic and energetic stress [24,25]. Accordingly, in MCF7/IGF2 cells we observed increased expression of two nuclear-encoded mitochondrial carrier proteins, the aspartate–glutamate mitochondrial carrier 1 (ARALAR/AGC1) and the pyrimidine nucleotide carrier 1 (PNC1/SLC25A33), as well as the nuclear respiratory factor 1 and 2 (NRF1/2), and mitofusin-1 (MFN1), which encodes for a protein involved in mitochondrial fusion (Figure 3C).

To further characterize the mitochondrial phenotype of MCF7/IGF2 cells, we demonstrated that MCF7/IGF2 presented increased mitochondrial activity, as assessed by measuring the levels of mitochondrial activity markers, such as nuclear factor erythroid-derived 2-like-2 (NFE2L2) transcription factor, which regulates the expression of anti-oxidant proteins, cytochrome C oxidase 1 (COX1), one of the three mtDNA encoded subunits of the respiratory complex IV and cytochrome B (cytoB), a component of the respiratory chain complex III (Figure 3D). Mitochondrial activity was also measured by MitoTracker Orange (MTO, 405 nm), which stains mitochondria in live cells upon oxidation (Figure 3E). Accordingly, MCF7/IGF2 cells had a slightly increased mitochondrial mass compared to control cells, as evaluated by assessing mRNA levels of translocase of outer membrane (TOMM20) and DNA copy number (Figure 3F). As mitochondrial mass reflects the balance between mitochondrial biogenesis and mitophagy [29], we investigated the mRNA expression of two mitophagy markers, the phosphatase and tensin homolog (PTEN)-induced kinase 1 (PINK1), a mitochondrial serine/threonine-protein kinase, and the B-cell lymphoma 2 (BCL2)/adenovirus E1B 19 kDa protein-interacting protein 3-like (BNIP3L), a mitophagy receptor protein induced by various stimuli [44,45]. While PINK1 was significantly increased by IGF2 expression, the levels of BNIP3L mRNA were not statistically different between MCF7/IGF2 and control cells (Figure 3G). Taken together these data strongly suggest that MCF7/IGF2 cells, in addition to high glycolytic activity, have also increased mitochondrial activity and slightly enhanced mitochondrial mass resulting from enhanced mitochondrial biogenesis partially balanced by increased rate of mitophagy. 

**Figure 4:**
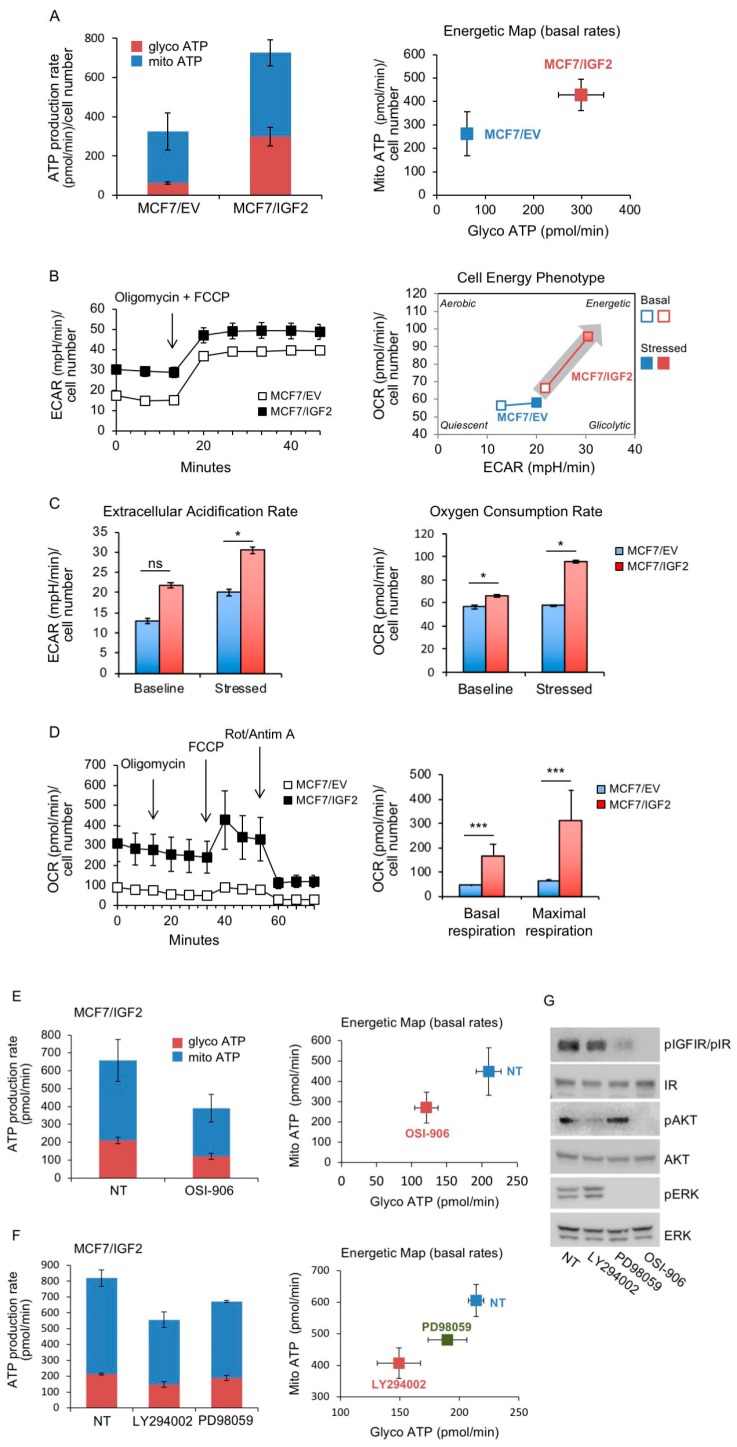
MCF7/IGF2 cells display increased ATP production both by glycolysis and oxidative phosphorylation (OxPhos). (**A**) ATP production rate in MCF7/IGF2 cells: the glycolytic ATP (glycoATP) and the mitochondrial (mitoATP) production rates were evaluated in MCF7/IGF2 cells as compared with MCF7/EV cells. Oxygen consumption rates (OCR) and extracellular acidification rates (ECAR) were first measured in basal conditions. Injection of oligomycin resulted in inhibition of mitochondrial ATP synthesis and decrease in OCR, allowing the mitoATP production rate to be quantified. Complete inhibition of mitochondrial respiration with rotenone plus antimycin A allowed accounting for mitochondrial-associated acidification and calculation of the glycoATP production rate according to the manufacturer instructions (Agilent ATP test). The histogram presented (left panel) and the energetic map (right panel) show the mean and range from two independent experiments. (**B**) Basal and stressed ECAR in MCF7/IGF2 cells in comparison to EV control cells. The simultaneous injection of oligomycin and carbonyl cyanide-4 (trifluoromethoxy) phenyldrazone (FCCP) caused a compensatory increase in the rate of glycolysis and drove the OCR higher as the mitochondria attempted to restore the mitochondrial membrane potential (Agilent phenotype test). (**C**) ECAR and OCR under basal and stressed conditions of cells treated as indicated in (B). Data presented are the mean and range from two independent experiments. (**D**) OCR was measured in MCF7/IGF2 cells and control cells by the XF cell mito stress test. Injections of respiration modulators including oligomycin, FCCP, rotenone and antimycin A were used to calculate key parameters of mitochondrial function (basal and maximal respiration are shown in the graph on the right). (**E, F**) Real time cell bioenergetics (Agilent ATP test) of MCF7/IGF2 cells was investigated in cells untreated (NT) and after exposure to the dual IR/IGF1R TK inhibitor OSI-906 (E), or the PI3K inhibitor LY294002 or the MEK1 inhibitor PD98059 (F). (**G**) Western blot analysis confirmed the effects of the inhibitors on pIGF1R/pIR, pAKT and pERK. (D–G) Values are means ± SEM of three independent experiments. (ns, not significant; * *p* < 0.05; *** *p* < 0.001).

3. Real time cell bioenergetics. The above results prompted us to test how glucose is utilized in IGF2 overexpressing cells. To this aim we assessed glycolysis and mitochondrial cell bioenergetics using the Seahorse XF^®^ technology. Our results confirmed the metabolic phenotype found in the glucose consumption/lactate production rate analysis. Indeed MCF7/IGF2 cells were significantly more metabolically active than control cells as total ATP production was enhanced by more than two-fold (726.25 pmol/min vs. 325.45 pmol/min in MCF7/IGF2 and MCF/EV, respectively; *p* = 0.05). The increase in ATP production was mostly due to enhanced glycolysis. ATP production from glycolysis was +475% higher in MCF7/IGF2 as compared to MCF7/EV cells (298.05 ± 47.05 pmol/min vs. 62.7 ± 5.7 pmol/min in MCF7/IGF2 and MCF7/EV cells, respectively, *p* = 0.019). At the same time, ATP from OxPhos in MCF7/IGF2 cells was +162% higher than in control cells (262.75 ± 94.4 pmol/min vs. 428.2 ± 66.2 pmol/min of ATP from OxPhos in MCF7/IGF2 and MCF7/EV cells, respectively, *p* = 0.144) (Figure 4A). In accordance with these results, MCF7/IGF2 cells showed a significant increase in both basal and maximal ECAR and OCR (Figure 4B,C) suggesting a switch towards a more energetic metabolic phenotype. In addition, in order to focus on the mitochondrial function we measured cell respiration using the Seahorse Mito Stress Test assay, that confirmed that both basal and maximal respiration were significantly increased in MCF7/IGF2 cells as compared to EV control cells (Figure 4D). Together, these results indicated that MCF7/IGF2 cells had an increased ability to enhance both glycolysis and OxPhos not only in basal metabolic conditions but also under metabolic stress induced by oligomycin and rotenone plus antimycin A injections. 

We next assessed the ATP production rate in MCF7/IGF2 cells incubated with 10 µM of OSI-906, a dual IR/IGF1R TK inhibitor, for 24 h (Figure 4E). We found that, in treated cells, total ATP production was reduced by −40.5% over untreated cells. However, this difference did not reach statistical significance (*p* = 0.083). Interestingly, ATP derived from glycolysis was instead significantly reduced in OSI-906 treated cells as compared to untreated ones (−42.1%; *p* = 0.011).

In order to elucidate the signaling pathways involved in MCF7/IGF2 cells bioenergetics, we also measured real time ATP production rates in cells exposed to 10 µM of phosphoinisitide 3-kinase (PI3K) (LY294002) or mitogen-activated protein kinase (MEK)1 (PD98059) inhibitors. Data showed that both inhibitors significantly reduced total ATP production: from 819.13 ± 55.9 pmol/min in untreated cells to 556.06 ± 54.2 pmol/min (*p* = 0.013) in LY294002 treated cells and to 670.70 ± 10.28 pmol/min (*p* = 0.029) in PD98059 treated cells. Of note, LY294002 reduced total ATP production more than PD98059 (−32.1% vs. –18.1%, *p* = 0.05). In addition, LY294002 reduced both glycolysis and mitochondrial derived ATP production by a similar rate (−30.3%, *p* = 0.014 vs. −32.7%, *p* = 0.024) as compared to untreated cells (Figure 4F). The effectiveness of OSI-906, LY294002 and PD98059 to inhibit respectively pIGF1R/pIR, pAKT and pERK was confirmed by Western blot analysis (Figure 4G). Overall, these data indicate that autocrine IGF2 strongly stimulates ATP production by enhancing both glycolysis and OxPhos, predominantly through the PI3K signaling pathway but, at a lower extent, also through the ERK1/2 pathway.

### 3.2. Establishment of MCF7 Cells Lacking IGF1R and Overexpressing IR-A

**Figure 5:**
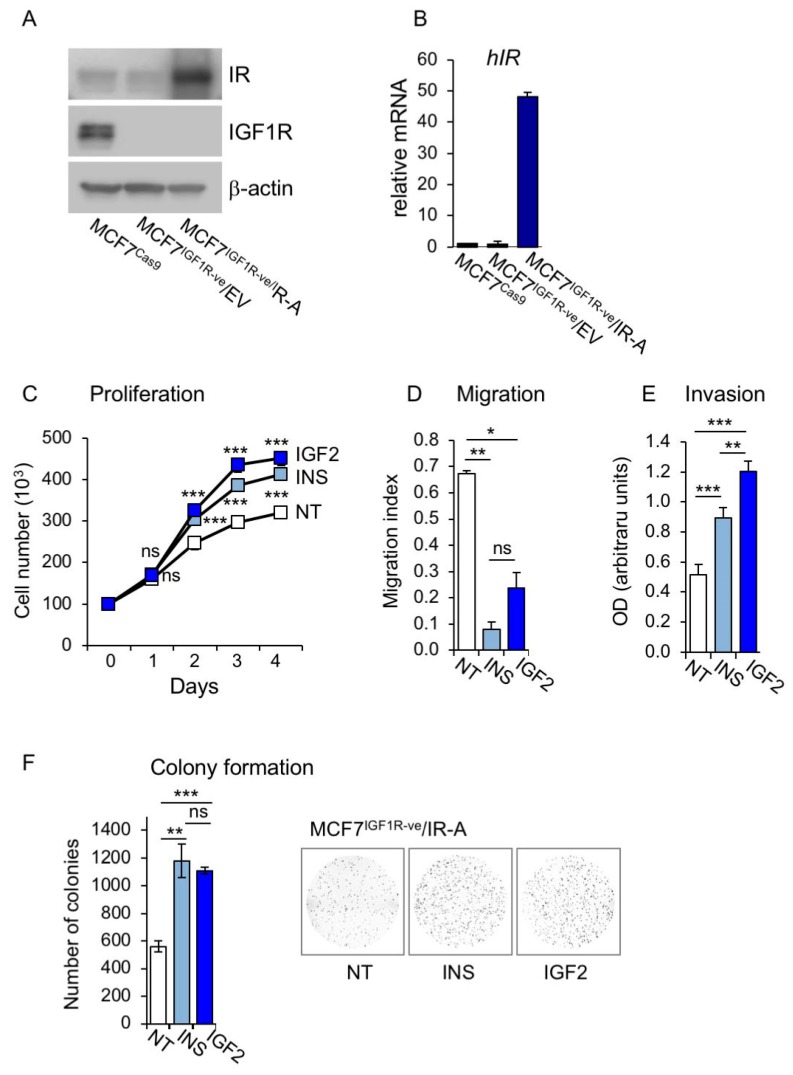
Characteristics and biological behavior of MCF7^IGF1R-ve^/insulin receptor isoform A (IR-A) cells. (**A**) MCF7^IGF1R-ve^/IR-A, control MCF7^IGF1R-ve^/EV cells and parental MCF7/CRISPR associated protein 9 (Cas9) cells were grown in 10% FBS, lysed and analyzed by SDS-PAGE and immunoblot for IR and IGF1R expression. (**B**) The same cells as in (A) were then analyzed for IR expression by qRT-PCR using human β-actin as the housekeeping control gene for normalization. Results are shown as means ± SE of three independent experiments. (**C**) Cell proliferation. MCF7^IGF1R-ve^/IR-A cells were cultured in the absence (NT) or presence of 10 nM of insulin or IGF2, and cell number measured by trypan blue exclusion assay at different time points, as indicated. (**D**) Cell migration was measured in a wound healing assay. MCF7^IGF1R-ve^/IR-A cells were seeded in six-well plates and allowed to reach confluence. After 24 h, we generated wounds in the confluent monolayers and incubation in serum free medium was continued for further 24 h in the absence (NT) or presence of 10 nM insulin or IGF2. Randomly chosen wound fields were photographed under a microscope every 8h for 24 h. The histogram represents the mean of the migration index calculated as follows: wound area after the indicated period/initial wound area. Representative fields are shown in the right panels. Experiments were performed in triplicates and data calculated as means ± SEM. Statistical significance of differences was analyzed using the Student’s t test. (**E**) Cell invasion. Serum starved MCF7^IGF1R-ve^/IR-A cells seeded on polycarbonate filters coated with 25 µg/mL fibronectin were then allowed to migrate for 6 h to the lower chamber in the absence (NT) or presence of 10 nM of insulin or IGF2. Values are means ± SEM of three independent experiments done in duplicate. (**F**) Colony formation. MCF7^IGF1R-ve^/IR-A cells were seeded in soft-agar and grown in serum free medium for three weeks in the absence (NT) or presence of 10 nM of insulin or IGF2. Colonies were stained with MTT and then photographed. The histogram on the right represents the number of colonies (means and range). Data shown are from two independent experiments run in quadruplicate wells. (ns, not significant; * *p* < 0.05; ** *p* < 0.01; *** *p* < 0.001).

To better define the metabolic effect of IR-A, which is the receptor principally involved in IGF2 action in cancer cells, we used MCF7 cells where the IGF1R was stably knocked-out by CRISPR/Cas technology and stably overexpressed the IR-A (MCF7^IGF1R-ve^/IR-A). As expected, MCF7^IGF1R-ve^/IR-A cells lacked the IGF1R and showed high IR levels (Figure 5A,B). When exposed to either insulin or IGF2, MCF7^IGF1R-ve^/IR-A cells showed enhanced proliferation, migration, invasion through fibronectin filters and colony formation (Figure 5C–F). Interestingly, both ligands were equipotent in stimulating cell proliferation and colony formation, while IGF2 was more potent than insulin in stimulating cell migration (Figure 5C–F). 

### 3.3. Metabolic Effects of Insulin and IGF2 in MCF7^IGF1R-ve^/IR-A Cells

**Figure 6:**
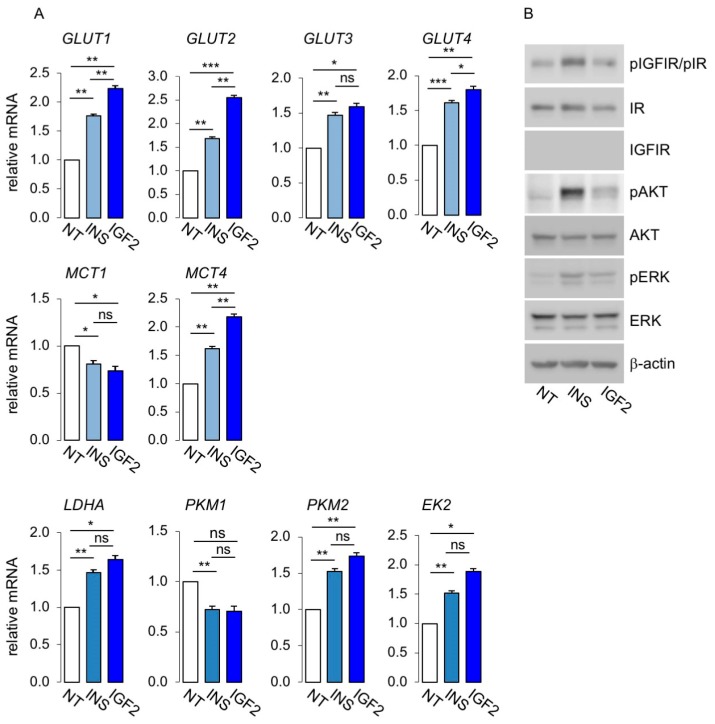
Expression of glucose and lactate transporters and glycolytic enzymes in MCF7^IGF1R-ve^/IR-A cells. (**A**) Cells were cultured in 10% charcoal stripped-FBS for 48 h, exposed to 10 nM insulin or IGF2 for 20 h or left untreated (NT), and then processed for mRNA expression for glucose and lactate transporters and glycolytic enzymes by qRT-PCR, as indicated. GAPDH was used as the housekeeping control gene. Values are means ± SEM of three separate experiments. (**B**) MCF7^IGF1R-ve^/IR-A cells, cultured as in (A) and exposed to either insulin or IGF2 or untreated (NT) were lysed, analyzed by SDS-PAGE and immunoblotted with the indicated primary antibodies. Phosphorylation of IGF1R/IR, AKT and ERK1/2 was evaluated to assess signal activation. β-actin was used as control for protein loading. Blots are representative of three independent experiments. (ns, not significant; * *p* < 0.05; ** *p* < 0.01; *** *p* < 0.001).

Next, we analyzed in MCF7^IGF1R-ve^/IR-A cells key molecules of glycolytic metabolism and mitochondrial biogenesis and activity by qRT-PCR and/or Western blot. As shown in Figure 6A, stimulation with both insulin and IGF2 increased the expression of all four glucose transporters evaluated (GLUT1 to GLUT4) and of lactate efflux transporter MCT4, while MCT1 was reduced. Glycolytic enzymes LDHA, PKM1, PKM2 and hexokinase-2 also increased after ligand stimulation. Overall, IGF2 elicited more robust effects than insulin. Phosphorylation of IGF1R/IR, AKT and ERK1/2 was evaluated by Western blot analysis to assess signal activation (Figure 6B).

**Figure 7:**
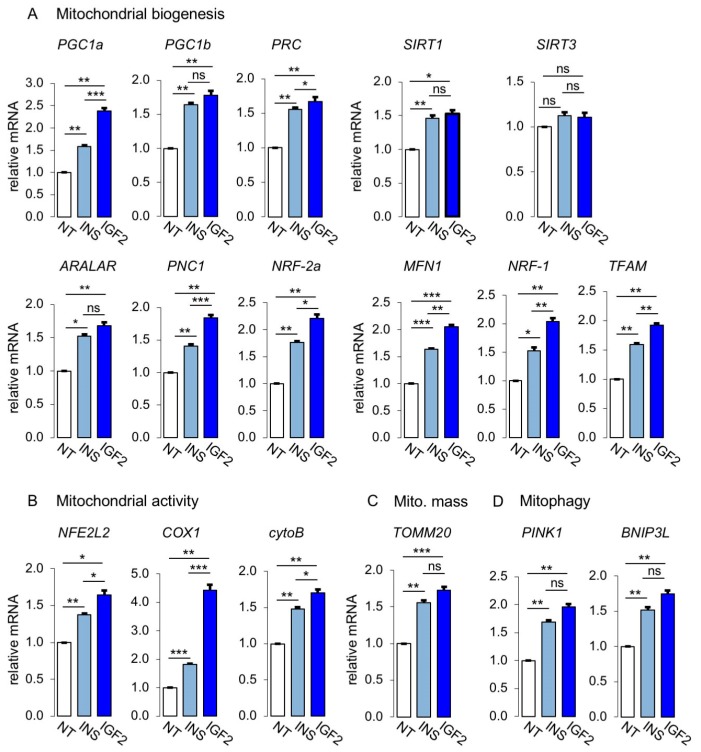
Mitochondrial activity was regulated at multiple levels by IR-A stimulation. mRNA expression levels of markers of mitochondrial biogenesis (**A**), mitochondrial activity (**B**), mitochondrial mass (**C**) and mitophagy (**D**), were determined by qRT-PCR in MCF7^IGF1R-ve^/IR-A cells stimulated or not (NT) with either insulin (INS) or IGF2 (10 nM for 20 h). Results are means ± SE of three independent experiments. (ns, not significant; * *p* < 0.05; ** *p* < 0.01; *** *p* < 0.001).

To elucidate the contribution of IR-A activation to mitochondrial activity, we analyzed the expression of markers of mitochondrial biogenesis and activity in MCF7^IGF1R-ve^/IR-A cells after stimulation with physiological concentrations of insulin or IGF2 at 10 nM. As shown in Figure 7A, markers of mitochondrial biogenesis were upregulated by both insulin and IGF2. Similarly, markers of mitochondrial activity (Figure 7B), mitochondrial mass marker, TOMM20 (Figure 7C), and mitophagy markers, PINK1 and BNIP3L (Figure 7D), were also upregulated after ligand stimulation. Taken together, these data strongly suggested that insulin and IGF2 by activating the IR-A stimulated not only glycolytic activity, but also mitochondrial biogenesis and activity as well as mitophagy. 

**Figure 8:**
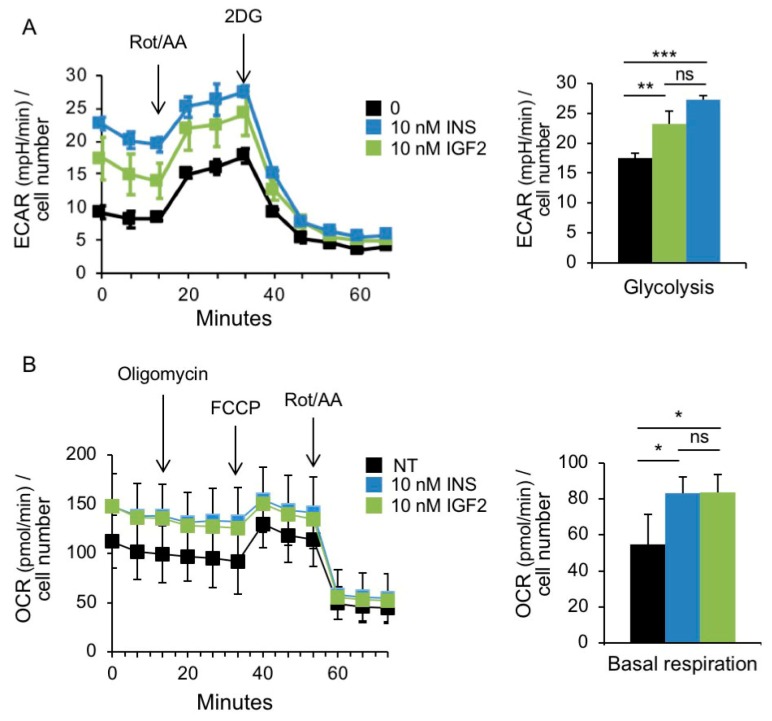
Insulin and IGF2 stimulate the metabolic activity of MCF7^IGF1R-ve^/IRA cells. (**A**) Glycolytic rate assay. Analysis of glycolysis rate (ECAR) after serial injections of metabolic modulators (a mix of rotenone and antimycin A and 2-deoxyglucose (2DG)) as indicated, in cells serum starved for 4 h and stimulated with 10 nM insulin or IGF2 for further 20 h or left untreated (NT). The bar chart on the right shows basal glycolysis. (**B**) Mitochondrial stress test. OCR was determined in cells treated as in (A) over a course of two hours in basal conditions and following the addition of the indicated uncouplers. The bar chart on the right shows the respiration. (A–B) Data are presented as means ± SEM of three independent experiments. (ns, not significant; * *p* < 0.05; ** *p* < 0.01; *** *p* < 0.001).

We analyzed glycolysis and mitochondrial bioenergetics in MCF7^IGF1R-ve^/IR-A cells in the absence or presence of insulin and IGF2 stimulation (Figure 8A,B). Real-time measurements of ECAR showed significantly higher basal ECAR in cells stimulated with either insulin or IGF2 (Figure 8A). When mitochondrial respiration was suppressed by injecting rotenone/antimycin A, glycolysis was stimulated to compensate for lack of energy production and this stimulation (glycolytic reserve) was also higher in cells exposed to both insulin and IGF2 (Figure 8A). Similarly, we assessed the effects of insulin and IGF2 stimulation on mitochondrial respiration (Figure 8B). Cells stimulated with both insulin and IGF showed higher OCR and ATP linked respiration as compared to untreated cells. This difference was maintained after treatment with metabolic inhibitors, as indicated (Figure 8B). Taken together, these data indicated that, in MCF7^IGF1R-ve^/IR-A cells, insulin and IGF2 stimulated both glycolysis and OxPhos, both in basal and high energy demand conditions. 

### 3.4. In BC Cells, Combination Treatment with Inhibitors of Glycolysis and Mitochondrial Respiration Has Additive Inhibitory Effects on IR-A Driven Migration and Colony Formation 

**Figure 9:**
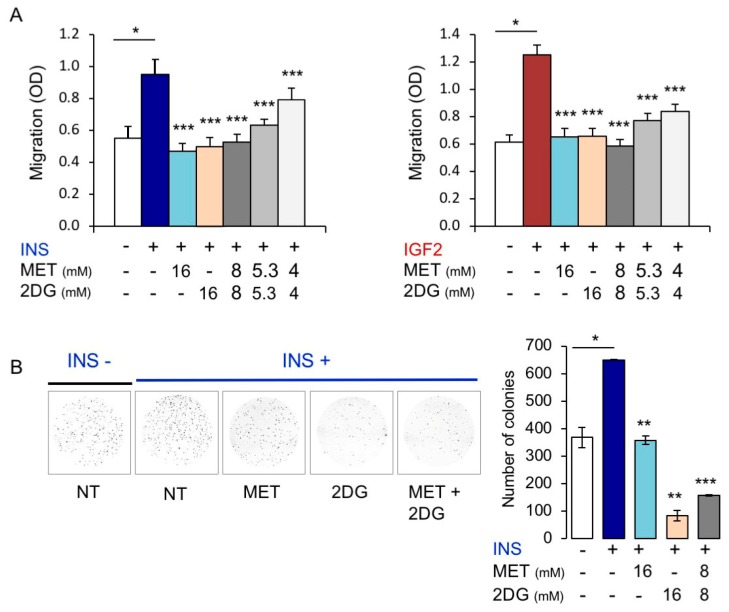
Metabolic inhibitors negatively regulated insulin (INS) or IGF2-dependent biological responses of MCF7^IGF1R-ve^/IRA cells. (**A**) Cell invasion. Serum starved MCF7^IGF1R-ve^/IRA cells were seeded on polycarbonate filters coated with 25 µg/mL fibronectin and treated with either metformin (MET) or 2DG alone or in combination. Cells were then allowed to migrate for 6 h to the lower chamber in the absence (NT) or presence of 10 nM INS or IGF2. Values are means ± SEM of three independent experiments done in duplicate. Asterisks indicate statistical significance vs. INS or IGF2 treated condition in the absence of metabolic inhibitors (* *p* < 0.05; *** *p* < 0.001). (**B**) Colony formation. MCF7^IGF1R-ve^/IR-A cells were seeded in soft-agar and treated with either MET or 2DG alone or in combination, in the absence (NT) or presence of 10 nM INS (see Methods). Colonies were then stained with MTT and photographed. The histogram on the right represents the number of colonies (means and range). Data shown are from two independent experiments run in quadruplicate wells. Asterisks indicate statistical significance vs. INS treated condition in the absence of metabolic inhibitors. (* *p* < 0.05; ** *p* < 0.01; *** *p* < 0.001).

Having observed that stimulation of MCF7^IGF1R-ve/^IR-A cells by insulin or IGF2 increased both glycolysis and mitochondrial respiration, we tested the effects of 2-deoxyglucose (2DG, inhibitor of glycolysis) and metformin (MET, inhibitor of mitochondrial complex I) on IR-A-driven biological responses. As shown in Figure 9A, physiological concentrations (10 nM) of either insulin or IGF2 strongly enhanced migration of MCF7^IGF1Rve-^/IR-A cells. Notably, ligand-induced migration of MCF7^IGF1Rve-^/IR-A cells was significantly reduced by both MET and 2DG at a dose of 16 mM. When cells were subjected to a combined treatment with MET plus 2DG, we observed an additive effect in inhibiting cell migration. Similar results were observed when we analyzed colony formation after insulin stimulation (Figure 9B). However, the number of colonies was more sensitive to 2DG than to MET inhibition. 

## 4. Discussion

In this study we demonstrated that the IGF2/IR-A axis elicits significant effects on metabolic reprogramming of human BC cells where it increases both glycolysis and OxPhos not only in basal conditions but also in high energy conditions demand, thereby increasing cancer cell metabolic flexibility, a key feature of malignant cells during adaptation to a changing microenvironment. 

*IGF2* is an imprinted gene, and loss or relaxation of imprinting enhances autocrine IGF2 levels and secretion in a variety of malignant cells, including BC cells [46,47,48]. Oncogene mutations and other molecular abnormalities leading to STAT3 activation also induce IGF2 secretion [49,50]. Additionally, IGF2 is produced by tumor stromal cells including cancer associated fibroblasts (CAFs) and macrophages [51,52,53,54]. IGF2 has similar binding affinity for IGF1R and IR-A, both of which are variably overexpressed in BC cells [55,56,57,58]. However, because the IGF1R has a higher binding affinity for IGF1 than IGF2, the IR-A is the main IGF2 receptor in an environment rich in both IGF2 and IGF1 [16,17,18]. Moreover, in insulin-resistant patients, the IR-A overexpressed in cancer cells is likely chronically activated by high circulating insulin levels [2,10,12]. Overall, IR-A overexpression in cancer cells has been implicated in tumor promotion, metastatic spread, stem-like cell phenotype, dedifferentiation and resistance to cancer therapies [1,2]. We hypothesized that the insulin/IGF2-IR-A axis might have also a role in the metabolic reprogramming of BC cells. 

First, we studied MCF7 human BC cells stably engineered to secrete IGF2. MCF7/IGF2 cells showed stronger capacity to proliferate, migrate and form colonies in association with higher metabolic activity as compared to control MCF7/EV cells. Notably, this increase in metabolic activity was mostly due to enhanced glycolysis but also to mitochondrial respiration. Both activities further increased when cells were subjected to high-energy demand by exposing them to oligomycin and FCCP. Consistent with increased aerobic glycolysis, MCF7/IGF2 cells consumed glucose more rapidly and produced more lactate as compared to control cells. They concomitantly showed an increase of glucose transporters GLUT1, GLUT2, GLUT3 and GLUT4, glycolytic enzymes LDHA and PKM2, as well as lactate transporter MCT4, which extrudes lactate from the cell. 

Specifically, LDHA is often upregulated in neoplastic tissues and, through actively transforming pyruvate to lactate, ensures ATP production and NAD regeneration, both essential in supporting cancer cell proliferation. Increased LDHA activity causes enhanced lactate production and its consequent export in the extracellular environment favors the metastatic process [59,60,61]. PKM2 catalyzes the last reaction of glycolysis to obtain pyruvate and ATP. Its expression is an important metabolic signature of tumor cells as it is considered an embryonic and cancer specific isoform [62,63]. Of note, PKM2 induces the expression GLUT1 and LDHA, involved in glycolysis [64].

These results are consistent with previous studies indicating that insulin upregulates PKM2 expression while decreasing its activity. These changes were likely critical in promoting glucose uptake, lactate production, glycolytic pooling and macromolecular synthesis in HEPG2 hepatoblastoma cells exposed to insulin [65]. Other reports have demonstrated that IGF1 upregulates PKM2 and induces its nuclear translocation [66].

However, MCF7/IGF2 cells additionally showed increased expression of various markers of mitochondrial biogenesis, including PGC1β, a master gene controlling mitochondrial biogenesis, PRC, and Sirt1, a deacetylase and activator of PGC1α, also involved in mitochondrial biogenesis. Markers of mitochondrial activity were also increased in association with a slight but significant increase in mitochondrial mass and specific mitochondrial DNA as well as a concomitant increase of mitophagy markers. 

Taken together, these findings indicate that MCF7/IGF2 cells have acquired the ability to increase their metabolic activity both in basal conditions and under increased energy demand. Although these cells show higher glycolytic activity as compared to MCF7/EV cells, they also acquire higher mitochondrial activity characterized by limited increase in mitochondrial mass and enhanced mitophagy markers. Published data suggest that AKT activation promotes aerobic glycolysis in leukemia and glioblastoma cells without increasing total oxygen consumption or cell proliferation [67]. However, in MCF7/IGF2 cells, both the PI3K/AKT and the ERK1/2 cascades were constitutively activated, suggesting a more complex mechanism of regulation. Notably, inhibitors of both pathways significantly reduced total ATP production, although the PI3K inhibitor LY294002 was twice more effective than the MEK1 inhibitor PD98059 (−32.1% vs. −18.1% total ATP production, *p* = 0.05). These data indicate that constitutive autocrine IGF2 strongly stimulates ATP production through both glycolysis and OxPhos, involving predominantly the PI3K signaling pathway but also the ERK1/2 pathway. These data were confirmed by inhibiting IR/IGF1R activation by the dual TK inhibitor OSI-906, which effectively inhibited ATP production. Both LY294002 and OSI-906 tended to be marginally more effective in inhibiting ATP derived from glycolysis. It is worth mentioning that in colorectal cancer cell lines IGF2 overexpression regulates sensitivity and/or response to IR/IGF1R TKI [68]. Further studies are needed to establish whether in BC cells high IGF2 and/or IR-A expression might be suitable biomarkers for increased sensitivity to IR/IGF1R TKI [69]. 

To investigate more specifically the effects of IR-A in the absence of IGF1R, we used MCF7 cells knocked out for IGF1R by CRISPR/Cas technology that were subsequently stably infected to overexpress the IR-A (MCF7^IGF1R-ve^/IR-A). These cells were characterized by a very high IR-A:IR-B ratio. Because of their very low relative abundance, endogenous IR-B are likely totally engaged in IR-A/IR-B hybrids that behave biologically as IR-A homodimers [70]. 

As expected, MCF7^IGF1R-ve^/IR-A cells showed increased ability to proliferate, migrate, invade and form colonies as compared to control EV cells in response to both insulin and IGF2 stimulation. In these cells, IR-A stimulated metabolic reprogramming and metabolic flexibility after exposure to insulin or IGF2. Overall, exposure with either insulin or IGF2 promote enhanced expression of both glycolytic and mitochondrial respiration markers. Real time cell bioenergetics confirmed that ligand stimulated MCF7^IGF1R-ve^/IR-A were characterized by increased basal glycolysis and maximal glycolytic capacity as well as increased basal and maximal mitochondrial respiration. Interestingly, IGF2 was slightly more potent than insulin in stimulating both glycolysis and OxPhos related gene expression and in stimulating cell invasion. These data are reminiscent of previous studies indicating that in cells lacking *IGF1R* and expressing solely IR-A, insulin and IGF2, differentially regulate receptor trafficking [71] and recruit a different network of proteins to the activated IR-A [71], thereby differentially modulating downstream signaling [72] and the expression of several genes [19].

Consistently with these results, in ligand stimulated MCF7^IGF1R-ve^/IR-A cells, a glycolysis inhibitor (2DG) as well as an OxPhos inhibitor (metformin) both blocked cell invasion and colony formation with additive effects in the combined treatment. 

One possible limitation of our work is the fact that some relevant experiments have been carried out in a single BC cell line. We have used MCF7 cells as a model system because they have been extensively characterized for their proliferative and invasive response to insulin/IGF signaling (IIGFs) [73]. MCF7 cells bear a PIK3CA E545K mutation, which might contribute to their sensitivity to insulin/IGFs [74]. However, whether this PIK3CA mutation might affect the metabolic impact of IGF2 and/or IR-A is currently not defined. As previously reported, different BC cell lines may have different sensitivity to insulin/IGFs in terms of proliferation, migration and survival [73,75]. Therefore, it would be important to further assess the metabolic impact of IGF-2/IR-A signaling in a comprehensive panel of BC cell lines. In this context, preliminary data of our group suggest that IR-A may additionally play a metabolic role in murine TNBC cells 4T1 (unpublished data). Notably, it has been recently reported that IGF1 stimulation increases mitochondrial biosynthesis and turnover in a panel of BC cells, including MCF7 cells [76]. Although these authors have not studied IGF2 or IR-A, their findings nicely support the concept that have a metabolic impact in BC cells.

## 5. Conclusions

Our study identifies upregulation of IGF2 and IR-A in BC cells as novel non-mutational mechanism contributing to metabolic reprogramming and increased metabolic flexibility. These IR-A-dependent metabolic effects are responsive to both IGF2 and insulin and might play a role in BC progression in obese and/or diabetic patients with hyperinsulinemia. These data might open new therapeutic approaches for BC overexpressing the IR-A.

## Figures and Tables

**Table 1:** PCR primers used.

*Gene*	Primers
*β-actin*	Fw 5′-GACAGGATGCAGAAGGAGATCACT-3′ Rv 5′-TGATCCACATCTGCTGGAACCT-3′
*ARALAR*	Fw 5′-CGAGACATTCCCTTCTCTGC-3′ Rv 5′-GTCCCTTTCCAAAATGCTGA-3′
*GAPDH*	Fw 5′-ACCCACTCCTCCACCTTTG-3′ Rv 5′-CTCTTGTGCTCTTGCTGGG-3′
*GLUT1*	Fw 5′-ATCGTGGCCATCTTTGGCTTTGTG-3′ Rv 5′-CTGGAAGCACATGCCCACAATGAA-3′
*GLUT2*	Fw 5′-AGCTGCATTCAGCAATTGGACCTG-3′ Rv 5′-ATGTGAACAGGGTAAAGGCCAGGA-3′
*GLUT3*	Fw 5′-AGCTCTCTGGGATCAATGCTGTGT-3′ Rv 5′-ATGGTGGCATAGATGGGCTCTTGA-3′
*GLUT4*	Fw 5′-TCGTGGCCATATTTGGCTTTGTGG-3′ Rv 5′-TAAGGACCCATAGCATCCGCAACA-3′
*Hexokinase2*	Fw 5′-TTGGCTTTTGCTTGGCAGAG-3′ Rv 5′-TCTTAATAGGGCCAAGCTCAGC-3′
*IGF2*	Fw 5′-GACCGCGGCTTCTACTTCAG-3′ Rv 5′-AGAACTTGCCCACGGGGTAT-3′
*IR isoform*	Fw 5′-CCAAAGACAGACTCTTCAGAT-3′ Rv 5′-AACATCGCCAAGGGACCTGC-3′
*IR total*	Fw 5′-CGTGGAGGATAATTACATCGTGTT-3′ Rv 5′-TGGTCGGGCAAACTTTCTG-3′
*LDHA*	Fw 5′-CATGGCAGCCTTTTCCTTAG-3′ Rv 5′-ATGACCAGCTTGGAGTTTGC -3′
*MCT1*	Fw 5′-GTGGAATGCTGTCCTGTCCTC-3′ Rv 5′-TCGATAATTGATGCCCATGCC-3′
*MCT4*	Fw 5′-ATTGGCCTGGTGCTGCTGATG-3′ Rv 5′-CGAGTCTGCAGGAGGCTTGTG-3′
*MFN1*	Fw 5′-TGTTTTGGTCGCAAACTCTG-3′ Rv 5′-CTGTCTGCGTACGTCTTCCA-3′
*b2 microglobulin*	Fw 5′-TGCTGTCTCCATGTTTGATGTATCT-3′ Rv 5′-TCTCTGCTCCCCACCTCTAAGT-3′
*NRF1*	Fw 5′-CGCTCTGAGAACTTCATGGAGGAACAC-3′ Rv 5′-GCCACATGGACCTGCTGCACTT-3′
*NRF2*	Fw 5’-AACAAGAACGCCTTGGGATAC-3′ Rv 5’-GTGAGGTCTATATCGGTCATGCT-3’
*PGC1α*	Fw 5’-CAAGCCAAACCAACAACTTTATCTCT-3′ Rv 5’-CACACTTAAGGTGCGTTCAATAGTC-3′
*PGC1β*	Fw 5’-GCTCAAGCTCTGGCTCTTCA-3′ Rv 5’-ATGCTTGGCGTTCTGTCTGA-3’
*PKM1*	Fw 5′-TGAAGAACTTGTGCGAGCCT-3′ Rv 5′-GCCAGACTCCGTCAGAACTA-3′
*PKM2*	Fw 5′-TTACCAGCGACCCCACAGAA-3′ Rv 5′-GACGATTATGGCCCCACTGC-3′
*PNC1*	Fw 5′-GCTCTGCAGCTTTTATCACAAATTC-3′ Rv 5′-AACGTAACGAGCACACTGGAGTG-3′
*PRC*	Fw 5′-CAAGCAGAAACAGAAGAGAGAAG-3′ Rv 5′-GGTGGGATGACAAGACAAGG-3′
*tRNAleu (UUR) mtDNA*	Fw 5′-CACCCAAGAACAGGGTTTGT-3′ Rv 5′-TGGCCATGGGTATGTTGTTA-3′
*hRP-S9*	Fw 5′-CTGGGTTTGTCGCAAAACTT-3′ Rv 5′-GTGGGTCCTTCTCATCAAGC-3′
*TFAM*	Fw 5’-ACTGCGCTCCCCCTTCAG-3′ Rv 5’-ACAGATGAAAACCACCTCGGTAA-3′
*TOMM20*	Fw 5′-GCTGGGCTTTCCAAGTTACC-3′ Rv 5′-TGTCAGATGGTCTACGCCCT-3′

Fw, forward; Rv, reverse.
